# Dosing Accuracy of Oral Extemporaneous Suspensions of Antibiotics: Measuring Procedures and Administration Devices

**DOI:** 10.3390/pharmaceutics13040528

**Published:** 2021-04-10

**Authors:** Inês Neves, Maria D. Auxtero

**Affiliations:** CiiEM, Interdisciplinary Research Centre Egas Moniz, Instituto Universitário Egas Moniz, Quinta da Granja, Monte de Caparica, 2829-511 Caparica, Portugal; ines_neves21@hotmail.com

**Keywords:** extemporaneous suspensions, administration devices, pediatrics, drug administration error

## Abstract

Extemporaneous suspensions are often marketed with several administration devices that can be freely used by patients/caregivers. The homogeneity of suspensions requires shaking before use. Hence, it is crucial to assess the precision of all devices and the users’ awareness of the shaking procedure. This study was conducted at University Institute Egas Moniz with 40 pharmacy students who were asked to measure 2.5 and 5 mL of two extemporaneous azithromycin suspensions. Formulation A is marketed with a double-dosing spoon and oral syringe, whereas B includes a transparent dosing spoon. Both have a reconstitution cup. The user’s preference for administration devices, the degree of compliance with the ‘shake before use’ instruction and the accuracy of the manipulation were assessed. The double-dosing spoon was the preferred device. The “shake before use” instruction was overlooked by most volunteers. The average measured volumes obtained with the double-dosing spoon were significantly different from the ones obtained with the oral syringe (*p* < 0.001) and significantly lower than the reference dose (*p* < 0.001). The oral syringe originates significantly higher values than the reference dose (*p* < 0.001). The dosing spoons values were significantly different from each other (*p* < 0.001). Liquid medicines containing several administration devices may be a challenge since they are nonequivalent.

## 1. Introduction

Children are at greater risk of medication errors [1] because (a) doses are calculated based on a patient’s age, weight or body surface area [2,3] and need readjustment as those parameters change; (b) inadequate availability of appropriate dosage forms and concentrations; (c) immaturity of defense systems, such as hepatic metabolism and other pharmacokinetic parameters that are markedly different from the adult population and may have a significant impact on pharmacological therapy. These factors must be considered to promote the correct and safe use of medicines in pediatrics [3]. Another important aspect of pediatric therapy concerns the need for adult caregivers (mainly parents, but also grandparents, nannies, health professionals, among others) to administer the medication to children who are unable to do it by themselves.

The most common type of medication errors are dosing errors, which often involve up to 10 times the actual dose required and antibiotics are among the most common classes of drugs associated with errors, probably because they are frequently prescribed [4,5]. Indeed, childhood medication often requires the use of lyophilized powder antibiotics meant to be extemporaneously reconstituted as oral suspensions. The antibiotics prescribed in pediatrics are mostly liquid oral formulations (particularly solutions and suspensions) due to the children’s inability to swallow tablets or capsules, the unavailability of certain antibiotics in the form of chewable tablets and the discomfort and cost associated with injectable formulations [6]. In addition, liquid formulations allow for easy adjustment of the dose depending on the child’s weight, and therefore individualize the doses. In these dosage forms, dose measurement is performed using administration devices, which may be vulnerable to dosing errors. The drug efficacy and effectiveness depend on the administration of the correct dose. Thereby, the safe and effective use of antibiotics in liquid oral suspension includes the correct reconstitution, concentration, ‘shake before use’ procedure, dose measurement, duration of treatment, and storage conditions [6,7].

In recent years, the resistance to antibiotics has increased, which is the ability that a microorganism develops to resist the effect of one or more drugs used to treat the pathologies caused by them. This can result in serious health consequences for the patient and is a major public health issue [8]. Misuse of antibiotics or incorrect dosing can contribute to bacterial resistance.

The choice of liquid formulations, particularly suspensions, has some disadvantages, including their poor homogeneity, unpredictable bioavailability of extemporaneous preparations, and the administration device’s accuracy that comes with the medicine [7].

The accuracy of the measurement performed by the patient/caregiver, especially in liquid oral pharmaceutical forms in pediatrics, is crucial to ensure the correct administration of the dose [9]. Using the proper administration device is essential for a more accurate dose and a uniform measurement [10]. In addition, for correct compliance to therapy, it is relevant that the dosing device is easy and practical to use from the patients’ perspective [11]. However, the administration of liquid oral formulations is susceptible to dosing errors mainly associated with the use of administration devices [12]. 

In Europe, the oral syringe is the most used dosing accessory, followed by the measuring spoon [13,14]. Nevertheless, several studies have reported that a measuring spoon does not guarantee the desirable dose [11,15,16]. Many liquid formulations available on the market include more than one type of administration device and the choice is often left to the patient’s preferences. In the case of medication for children, the choice is left to the caregiver that administers the dose and that can be a different person in different administration times.

In general, the use of calibrated administration devices should ensure an accurate and precise measurement of the dose [9] and promote an adequate therapeutic response by making a correct and simple administration, avoiding overdosing or under-dosing and reducing errors in drug administration.

Another important aspect of oral liquid suspensions refers to the ‘shake before use’ procedure, which is a key point for the homogeneity of the formulation, and, therefore, for a correct dose administration [17].

Some works have been reported on the accuracy of administration devices [6,9,10,18], but none discuss the importance of the design of a given type of device neither the risk of misuse of reconstitution devices as administration devices. The compliance of shaking suspensions before use was assessed through a cross-sectional questionnaire applied to mothers but not experimentally evaluated [6]. Hence, the present work aims to compare the accuracy of the administration devices included in the package of an antibiotic for extemporaneous suspension; to compare different models of the same device (two designs of spoon supplied with different formulations); to assess the preference of young adult users for the different types of devices supplied with the antibiotic and, to evaluate the compliance with the label instructions as to the need of ‘shaking before use’.

## 2. Materials and Methods

### 2.1. Subjects

The experimental study was conducted at University Institute Egas Moniz, with 40 randomly selected pharmacy students from a population of 138 students, based on the following selection criteria:

▪ Inclusion criteria: Student of Pharmaceutic Sciences Master course aged 18 or more years old.
▪ Exclusion criteria: Volunteers who do not complete the measurement plan.

The choice of pharmacy students as volunteers had a double purpose: as potential future young parents on the caregivers’ role for their children and as future health professionals. The study group was constituted by 32 women (80%) and 8 men (20%), with an average age of 21.5 years old, ranging from 18 to 32 years old. Most of these students (65%) had already taken Pharmaceutical Technology and/or Galenic Pharmacy courses (Table 1) when this study was conducted. The protocol of the study was approved by the Egas Moniz Ethics Committee (protocol code nº 764; 26 June 2019). Informed consent was obtained from all the participants and personal data was collected, including age, gender and diagnosed eyesight problems (Table 1). 

The eyesight problems were registered to evaluate whether this condition influences the results obtained, especially in the measurement of the requested doses. Volunteers were asked about diagnosed eyesight problems and, in case of an affirmative answer, if they intended to use any kind of vision aid (lenses or glasses) at the moment of performing the measurements. The sample was divided into two groups, group 1 and group 2. Group 1 included the students with eyesight problems but not using any vision aids (*n* = 9). Group 2 was constituted by the volunteers with no eyesight problems and eyesight problems using lenses or glasses to measure the requested dose.

### 2.2. Materials

**Figure 1:**
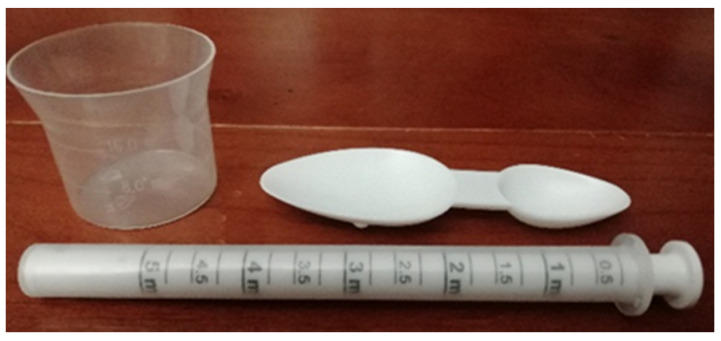
Reconstitution cup (volume marks = 8 and 16 mL), double-dosing spoon (volume = 2.5 and 5 mL) and oral syringe (volume = 0–5 mL) of the antibiotic A.

**Figure 2:**
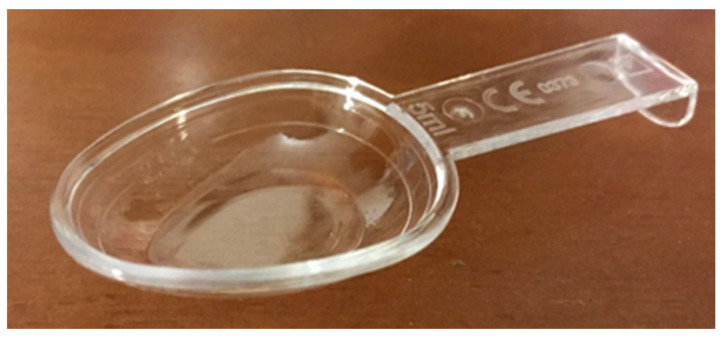
Transparent dosing spoon used for antibiotic B (volume marks at 2.5 and 5 mL).

In the present study, two extemporaneous suspensions of azithromycin (40 mg/mL) (A and B) were used as representative of antibiotics supplied with different administration devices available on the Portuguese pharmacy market. Antibiotic A included an opaque double-dosing spoon (capacity of 2.5 mL in one end and 5 mL in the other end, both without volume marks) and oral syringe (maximum capacity of 5 mL, with a mark in every 0.25 mL) as administration devices and one graduated reconstitution cup, with volume marks at 8 and 16 mL (Figure 1). Antibiotic B included a single transparent dosing spoon, with two volume marks at 2.5 and 5 mL (Figure 2) and an oral syringe as administration devices, as well as one graduated reconstitution cup. The reconstitution cup and the oral syringe of antibiotic B were not used in this study. All administration devices tested were the ones authorized and included in the original package of extemporaneous suspensions measured. For this study, an analytical weighing scale was used (Sartorius TE214S, d = 0.0001 g). The scale was calibrated every day using a weight of 200 g.

The researcher prepared both extemporaneous suspensions, using distilled water, following the instructions included in the package’s leaflets. In the case of Antibiotic A, the vial containing the powder for oral suspension was shaken, then 16 mL of distilled water measured using the reconstitution cup were added. For Antibiotic B, the procedure was the same, but the volume of water added was 15 mL. Participants were informed of the study’s objective and free access to the packages and leaflets was given to volunteers during the conduct of the experiment.

### 2.3. Conduct of the Experiment

Since there is a risk that user’s may misuse the reconstitution cup as a dosing device, in the first part of our experiment, volunteers were requested to rank the devices they would use to measure a dose if they had to take the medicine A or to administer it to someone else, starting with their favorite one. No information was provided about the three administration devices or their volumetric capacity, but free access to the information stated on the package was warranted.

Then, volunteers were requested to measure two doses of each antibiotic, corresponding to 5 and 2.5 mL, starting with antibiotic A and using both oral syringe and double-dosing spoon in the preferred order. Regarding antibiotic B, volunteers were requested to first measure 5 mL and then 2.5 mL, using the transparent dosing spoon. In all cases, devices were carefully rinsed and dried in between measurements.

Before the first measurement, we observed if the volunteers shook the antibiotic formulation, as stated on the label. Whenever they failed to shake it, they were prevented from measuring without fulfilling that requirement and were informed of the importance of this procedure. Then, we observed whether this information was understood and implemented by the volunteers in the following dose measurements.

Each volunteer made all the measurements in triplicate and in one day.

### 2.4. Measurements of Volume Accuracy

The 2.5 and 5 mL volumes of antibiotic A and antibiotic B were rigorously weighed using a 5000 μL micropipette (Gilson Incorporated, Middleton, WI, USA). The calibration of the 5000 μL micropipette was confirmed by measuring 5 mL of distilled water (d = 1 kg/m^3^). Reference weights were measured in triplicate.

Then, volumes measured by volunteers were weighed, including the measuring device, and the net weight of the measuring device was subtracted. The final weight was compared to the reference values.

Whenever measurements were performed using the oral syringe, volunteers deepened the device into the formulation to a maximum of approximately ¼ of the syringe length and the amount on the outer side of the wall was included in the analysis.

### 2.5. Statistical Analysis

Statistical analysis was perform using the IBM SPSS Statistics v.25.0 (IBM Corp., Armonk, NY, USA) for Windows and included descriptive measures such as the average and the standard deviation. In order to compare the average volume values, inferential statistical analysis methodologies were applied (Student’s *t*-Test: independent samples *t*-test, paired sample *t*-test and one sample *t*-test).

The application assumptions were verified and validated for each inferential procedure. The level of significance was 5% in all analyzes of comparative statistical inference.

## 3. Results

### 3.1. Reference Weights

The reference weights for the 2.5 and 5 mL volumes of antibiotics A and B are shown in Table 2.

### 3.2. Eyesight Problems

It has been observed that the eyesight problems did not influence the result in both measurement procedures of 2.5 and 5 mL (*p* = 0.068 to *p* = 0.966).

### 3.3. Preference Order of Administration Devices for Oral Administration of Antibiotic A

**Figure 3:**
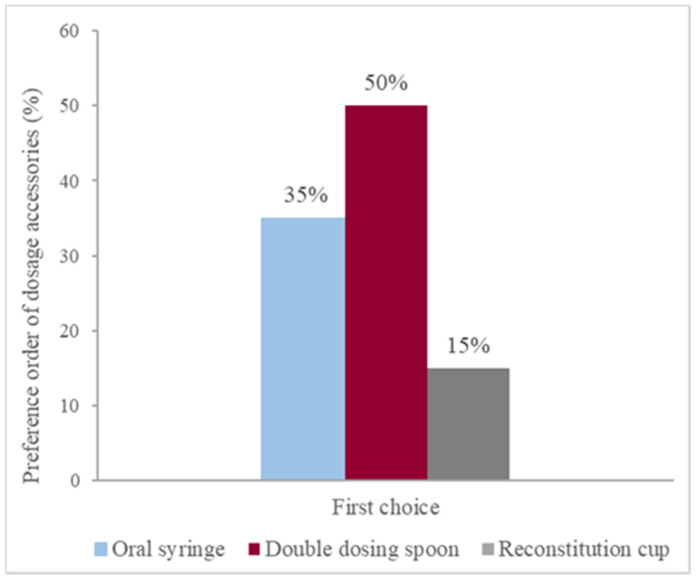
Administration devices selected as the first choice for antibiotic A administration.

Half of the volunteers (*n* = 20) selected the double-dosing spoon as their first choice of device for antibiotic administration, whereas 35% (*n* = 14) preferred an oral syringe and 15% preferred a reconstitution cup (*n* = 6) (Figure 3). Only twelve volunteers (30%) asked about the volume that was to be measured prior to the choice of the device.

Regarding the reconstitution cup, all volunteers verified the existence of two volume marks indicating the volumes that could be measured (8 and 16 mL). Nevertheless, five volunteers (13%) still tried to use the reconstitution cup to measure 5 mL.

### 3.4. Comparison between Double-Dosing Spoon and Oral Syringe of Antibiotic A

For antibiotic A, the average weights corresponding to the measured volumes of 2.5 and 5 mL are shown in Table 3 for both devices.

In the 2.5 mL and 5 mL volume measurements, the average weight values of the volumes measured with the double-dosing spoon were significantly lower than the average weight of the volume measured with the oral syringe.

### 3.5. Comparison between Double-Dosing Spoon of Antibiotic A and Transparent Dosing Spoon of Antibiotic B

For both volumes of 2.5 and 5 mL, significant differences in dose measurement were obtained using the double-dosing spoon or the transparent dosing spoon. The average weight values corresponding to both volumes measured using the two devices are shown in Table 4. In the case of the double-dosing spoon (Formulation A) the volumes of 2.5 and 5 mL were measured with the 2.5 and the 5 mL spoon ends, respectively. Regarding the measurements using the transparent dosing spoon (Formulation B) the volumes of 2.5 and 5 mL were measured following the 2.5 and the 5 mL spoon marks, respectively.

The average weight values measured by the transparent dosing spoon was a value significantly higher than the average weight measured with the double-dosing spoon for both volumes.

### 3.6. Comparison between Measurements Made with the Three Administration Devices and Reference Values

#### 3.6.1. Volume 2.5 mL

When measuring the volume of 2.5 mL the double-dosing spoon originated a significantly lower dose (2.3 (±0.4) g) than the reference value (3.18 (±0.01) g), whereas the oral syringe leads to a significant overdose (3.3 (±0.1) g). In both cases *p* < 0.001. In the case of the transparent dosing spoon of antibiotic B, the measured mean value of 3.4 (±0.4) g was significantly higher than the 3.23 (±0.01) g of the reference dose (*p* < 0.001).

#### 3.6.2. Volume 5 mL

Measuring a 5 mL volume with the double-dosing spoon, a significant underdose was obtained (5.0 (±0.7) g vs. 6.31 (±0.03) g) (*p* < 0.001). On the other hand, the oral syringe measured an average value of 6.5 (±0.2) g, significantly higher than the reference dose (6.31 (±0.03) g) (*p* < 0.001). For antibiotic B, the transparent dosing spoon measured a significantly higher average value of 7.6 (±1.1) g, compared to the 6.35 (±0.06) g of the reference dose (*p* < 0.001).

#### 3.6.3. Percentage Analysis of Administration Devices Deviations

When comparing the values of the volumes measured by the volunteers with the reference volume values, it was observed that double-dosing spoon originates underdosing. Furthermore, the deviation from reference values were higher for 2.5 mL (31.4%) than for 5 mL (24.4%).

**Figure 4:**
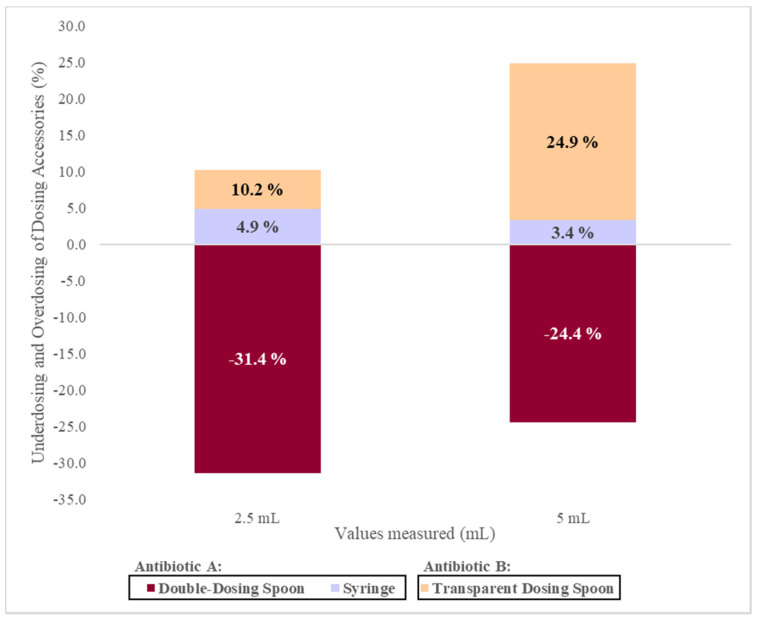
Percentage of deviation from the reference dose, using different administration devices.

On the other hand, with the transparent dosing spoon, an overdosing is observed with a greater impact on the volume of 5 mL (24.9%) when compared to the volume 2.5 mL (10.2%). Finally, with the oral syringe, 4.9% and 3.4% overdosing was also observed in the volumes of 2.5 and 5 mL, respectively (Figure 4).

### 3.7. Evaluation of “Shake before Use” Procedure

**Figure 5:**
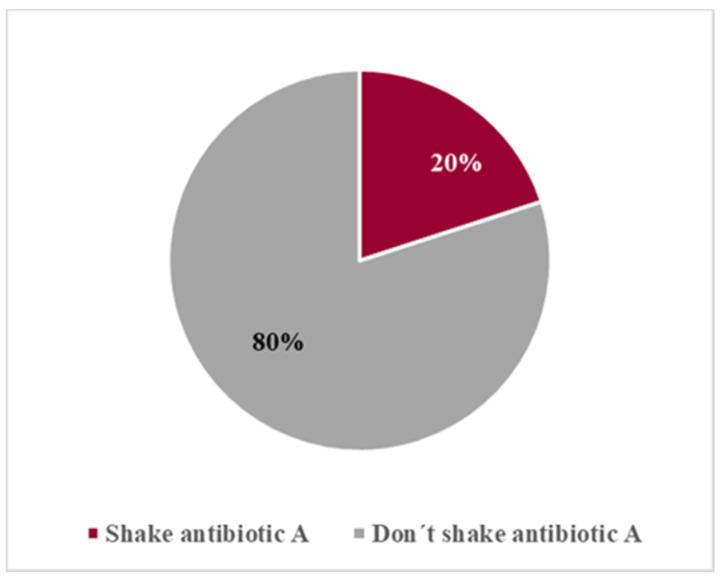
Assessment of compliance with “Shake before use” instruction for antibiotic A.

In the first contact with the oral suspension of antibiotic A, only eight volunteers (20%) shook it before use (Figure 5).

**Figure 6:**
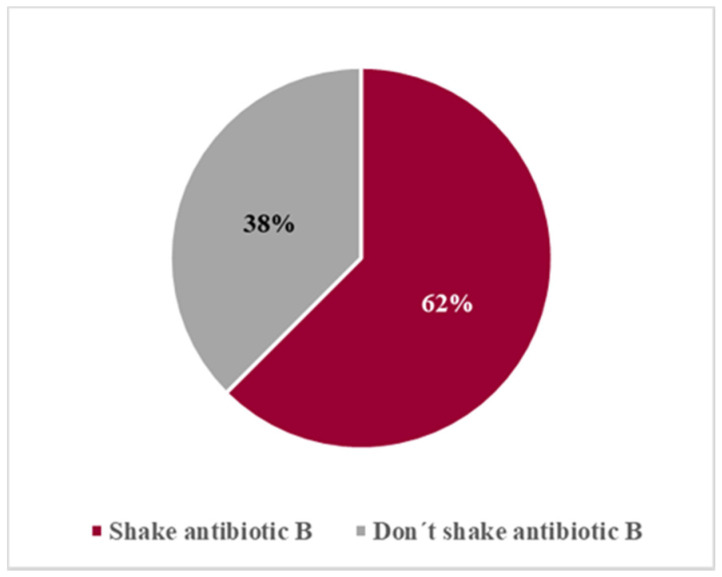
Assessment of compliance with “Shake before use” instruction for antibiotic B.

For the measurement of antibiotic B (the last one to be measured), 62% of the 40 volunteers (25 volunteers) remembered the importance of shaking the formulation before measuring the dose (Figure 6). Almost 50% of the volunteers failed to shake antibiotic A and failed again with antibiotic B (15/32), even though they had been informed of the importance of the procedure. Eight (8) of these 15 volunteers that never shook the antibiotics, were first- or second-year pharmacy students. However, in all measurements of both antibiotics A and B, volunteers who forgot to shake the oral suspension were instructed to do it before performing the measurement. Hence, all measurements were performed after the suspensions were properly shaken.

## 4. Discussion

This study demonstrates that measuring the correct dose of the suspension using the administration devices provided with the products may lead to inaccurate administration of dose and consequently the associated side effects. In this study, all suspensions were prepared by the researcher, following the leaflet instructions prior to dose measurements. Antibiotic A included one graduated reconstitution cup with two-volume marks set at 8 and 16 mL, and two administration devices (oral syringe and opaque double spoon). Despite knowing that the graduated cup was meant to measure either 8 or 16 mL, five volunteers tried to use it to measure 5 mL. This observation shows that inappropriate reconstitution devices provided with the products can increase the risk of incorrect use of the administration and/or reconstitution devices, and no previous work has been reported on this topic.

The average values measured with the double-dosing spoon (2.3 (±0.4) g for 2.5 mL and 5.0 (±0.7) g for 5 mL) are significantly lower than the reference values, which results in underdosing. On the other hand, the transparent dosing spoon originated average values of 3.4 (±0.4) g for 2.5 mL and 7.6 (±1.1) g for 5 mL that is significantly higher than the reference values, which promotes overdose. Previous independent studies have also presented different conclusions regarding the accuracy of the dosing spoon. Berthe-Aucejo et al. [16], found that the use of the dosing spoon generally promotes underdosing. Ryu and Lee [19] demonstrated that the average value measured with the dosing spoon is 4.6 (±0.6) mL for a volume of 5 mL. On the other hand, Yin et al. [20] concluded that to measure a volume of 5 mL, the average value obtained is 5.5 (±0.7) mL, which results in an overdose. However, these authors only studied one design variant of the dosing spoon, while in the present work, two different dosing spoon designs were evaluated.

In those studies, no considerations were made about the particularities of the spoon and it is impossible to establish a relationship between the model of the device and the nature of the observed dosage deviation. In the present study, it was clear that the design of the administration device plays an important role in the correct measurement. As said before, the opaque double-dosing spoon is made of fragile plastic material, does not have a volume mark and its limit capacity corresponds to the edge. Volunteers could not choose between the two types of spoons since an opaque double-dosing spoon was meant to measure antibiotic A and a transparent spoon was meant for antibiotic B. However, all of them reported difficulties in handling the opaque double-dosing spoon due to the absence of a handle and showed insecurity to fill the spoon without spilling the suspension due to the opacity that made it difficult to see the contents and the lack of volume marks. Hence, underdose is more likely to happen. 

**Figure 7:**
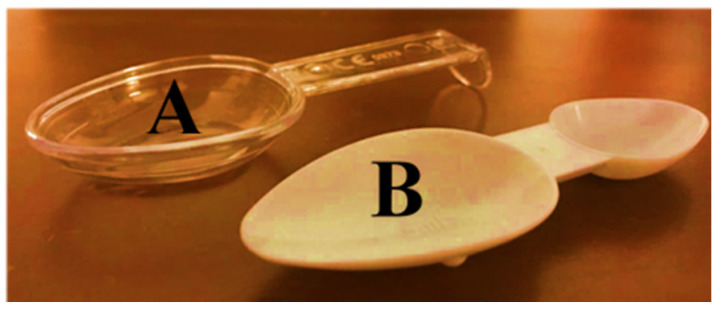
Comparison of design between the double-dosing spoon (under-dosing) and the transparent dosing spoon (overdosing). ((**A**) = With rim on periphery and Handle; (**B**) = Without rim on periphery).

On the other hand, the transparent dosing spoon is made of more resistant plastic, has a handle and includes two-volume marks (for 2.5 and 5 mL), both below the outer edge of the spoon (Figure 7). The use of a transparent device allows for better visualization of the volume marking when compared to an opaque device. These aspects can provide greater confidence to the volunteers since they are not afraid of spilling the suspension, and tend to over-measure. Nevertheless, if the marks on the spoon were colored, a better visualization could be possible. With this evidence, it is possible to conclude that the design of the administration device also significantly affects the accuracy of the measurement. The design of the administration device supplied in the medicine package could grant confidence or insecurity to the patient and/or caregiver at the moment of performing the measurement. To our knowledge, no previous works have discussed these design features and they are of utmost relevance to the pharmaceutical industry and must be considered when designing administration devices.

The mean values measured with the oral syringe (3.3 (±0.1) g for 2.5 mL and 6.5 (±0.2) g for 5 mL) are significantly higher than the reference value (2.5 mL = 3.18 (±0.01) g and 5 mL = 6.31 (±0.03) g) resulting in an overdose. However, previous studies have reported the use of oral syringes associated with greater accurate measurements [6,10,18]. This measurement error can be explained by the fact that the oral syringe does not have an adapter for the amber vial of the antibiotic. Therefore, to perform the measurement, it is necessary to insert the syringe into the bottle, which contributes to overdose, not necessarily caused by an incorrect measurement but due to some amount on the outer side of the wall, which was included in the analysis. Nevertheless, in order to minimize this problem, volunteers were requested to insert the syringe to a maximum of about one-fourth of the syringe length. This allows for a better comparison with real practice as children may insert a variable portion of the syringe in the mouth, but probably not more than ¼ of its length. The design of the specific syringe used in this study does not include a nozzle which could be the only part of the syringe to be inserted in the child’s mouth. A previous study with a similar methodology for 5 mL, also determined an average value higher than the reference value; however, no information was available regarding the design of the oral syringe [21].

It was found that there are significant differences in dose measurement using the double-dosing spoon and the oral syringe in the volume of 2.5 and 5 mL. The double-dosing spoon has a higher percentage of error than the oral syringe. These findings are in agreement with previously reported studies [9]. Additionally, antibiotics included more than one administration device leaving the choice to the user. As verified in our work, user preferences are variable and the double measuring spoon was the preferred accessory over oral syringe and reconstitution cup. However, during the utilization of the double-dosing spoon and the transparent spoon, volunteers clearly preferred the design and characteristics of the transparent dosing spoon. Previous works also assessed user preferences ranging from dosing spoon [9,10], oral syringe [6] to dosing cup [18]. These facts make it possible to speculate that any device available with the medicine will allow for identical doses. Eventually, the patient or caregiver can use more than one different device during the same therapy, including the hypothesis of using devices kept from other medications. This is particularly relevant in the case of pediatrics since the administration of medicines is carried out by caregivers (parents, grandparents, nannies) who may change during the treatment period, increasing the risk of use of different devices to measure each dose. Our study shows that the device used significantly affects the accuracy of the measured dose, even between devices of the same type (e.g., two dosing spoons). Of the three devices studied, the oral syringe has the lowest measurement error despite being significantly higher than the reference value, followed by the transparent dosing spoon and finally the double-dosing spoon. Previous studies also reported the oral syringe is the most accurate device [10,22].

On the other hand, the volume to be measured has no impact on the result obtained since there were significant differences between the devices in the dose measurement, both in the smallest (2.5 mL) and in the largest volumes (5 mL).

In our study, the characteristics of the suspensions (such as viscosity) were not determined and neither in most of the published works. The choice of formulations of the same antibiotic, in the same concentration and prepared in a similar way, aimed to minimize differences between the two suspensions.

It can be concluded that the administration device provided with oral liquid formulations can play a central role in medication errors. Even though several works have been published on these issues [6,10,18] to our knowledge, this is the first time that the design of a specific type of device (spoons) was considered as a key factor for an accurate measurement. Likewise, this is the first work evaluating the risk of misuse of reconstitution cups as administration devices and the variability of dose measurements arising from the existence of more than one device inside the medicine package, freely chosen and randomly used by users/caregivers. This issue is of great importance for all liquid formulations but has an increased impact in pediatrics due to the fact that children depend on caregivers to administer medications, and one child can have several caregivers during the treatment period.

Another important feature of liquid suspensions is the need for shaking prior to use in order to ensure homogeneity of the medicine. This type of formulation includes a warning message on the label ‘Shake before use’. Nevertheless, our study revealed that users do not always pay attention to the label instructions and, even when orally informed, they tend to forget the importance of the procedure. However, those with more difficulty to comply were students that had not taken courses related to medicine production (Galenic Pharmacy or Pharmaceutical Technology) and were unaware of the issue. Those can be considered as layperson, such as parents or other caregivers who are using the device and the medicine for the first time, and need to be instructed about the correct use of the devices and the need for shaking the suspension before use. A previous work demonstrated the benefit of provider use of advanced counseling strategies and dosing instrument provision in reducing errors when used together [23]. As a matter of fact, providing parents and caregivers with formation about proper handling of administration devices has proven important to avoid dosing errors [10,24].

## 5. Conclusions

The main objectives of the present work were to evaluate the accuracy of different administration devices supplied with oral liquid antibiotic suspensions, comparing different types of devices included in the same antibiotic package, as well as comparing two different models of spoon provided with two different formulations of the same antibiotic in the same concentration (azithromycin 40 mg/mL). Additionally, we aimed to assess the preferences of the user/future pharmacists towards those devices, including reconstitution cups, as well as the fulfillment of the shaking procedure before use, which is mandatory for correct dosing.

This study demonstrated that the oral syringe is the administration device with the smallest error in dose measurement, when compared to the dosing spoon provided in the same package. It was found that the administration devices provided with the medications do not guarantee the accuracy of the required dose and that overdosing and underdosing depend not only on the type of device but also on its design.

Oral administration of liquid dosage forms can be complex and difficult, especially in children, due to the inability to self-control the measurement and intake of the medication and, therefore, depending on several caregivers. In addition, the existence of a wide range of administration devices with different formats also contributes to variations in volume measurements.

Moreover, the inclusion of reconstitution cups in the packages of extemporaneous suspensions can lead to their misuse for dose measurement.

To ensure a correct dosage, it is important to advise caregivers on which device to use and explain how the measurement should be performed, as well as to reinforce the awareness of the user about the importance of shaking suspensions before measuring the dose.

Health professionals, like pharmacists, play an important role in these issues as they have close contact with patients and/or caregivers, and must teach them about the correct use of dosing devices and medicines in the act of dispensing the medication. To this end, good oral communication is essential, along with other strategies, such as the explanation with pictograms, clearly indicating the accessory to be used according to dose and age and whenever possible, showing how to use the devices. Regarding the reconstitution cup, since the preparation of the suspension is meant to be performed by the pharmacist at the pharmacy, the cup should be discarded after use. Alternatively, reconstitution cups could be printed with the indication ‘not meant for drug administration’ or similar. Additionally, pharmaceutical companies can include a step-by-step diagram instruction in the leaflet to demonstrate how to accurately use the administration devices [10].

All these measures may contribute to health promotion and to the rational use of medicines.

In brief, our work adds novelty in aspects such as the fact that the design of devices of the same type (spoon, in this case) is highly related to users’ insecurity in measuring the correct volume. Moreover, this work shows that the inclusion of different devices inside the package of a liquid formulation represents an extra uncertainty factor since different users may use different devices at a time for dose measurement, which can result in higher variability in dosages during the treatment period. This is of special concern in pediatrics, since children depend on one or several caregivers to administer the medicine. Additionally, to our knowledge, this is the first work that evaluates the risk of misuse of reconstitution cups as an administration device.

Finally, this study has some limitations, such as the fact that the deviations were evaluated in terms of weight and volumes of suspension, which do not necessarily reflect proportional variation in drug content. This aspect is to be considered and addressed in future work.

## Figures and Tables

**Table 1:** Characteristics of volunteers.

Volunteer Characteristics	Number (%)
Gender	
Female	32 (80%)
Male	8 (20%)
College year	
First year	5 (12.5%)
Second year	9 (22.5%)
Third year	11 (27.5%)
Fourth year	8 (20%)
Fifth year	7 (17.5%)
Eyesight problems	
Myopia	11 (27.5%)
Stigma	5 (12.5%)
Myopia and Stigma	4 (10%)
Hyperopia	1 (2.5%)
None	19 (47.5%)
Vision aids	
Glasses (Group 2)	6 (15%)
Lenses (Group 2)	6 (15%)
None (with eyesight problems) (Group 1)	9 (22.5%)
None (no eyesight problems) (Group 2)	19 (47.5%)

**Table 2:** Reference weights and respective standard deviation (SD) (*n* = 3), for the average value of volumes 2.5 and 5 mL of antibiotic A and antibiotic B.

Formulation	Average Weight (±SD) (g) *n* = 3
Antibiotic A	
Volume 2.5 mL (small end of the double spoon)	3.18 (±0.01)
Volume 5 mL (large end of the double spoon)	6.31 (±0.03)
Antibiotic B	
Volume 2.5 mL (lower mark of the transparent spoon)	3.23 (±0.01)
Volume 5 mL (upper mark of the transparent spoon)	6.35 (±0.06)

**Table 3:** Average weight (*n* = 40), standard deviation (SD) and 95% confidence interval (95% CI) for 2.5 and 5 mL of antibiotic A, measured with a double-dosing spoon and oral syringe.

Measured Volume	Average Weight (±SD) (g) *n* = 3	CI 95%	*p* ^1^
2.5 mL			
Small end of the double-dosing spoon	2.3 (±0.4)	(2.1–2.4)	<0.001
Oral syringe	3.3 (±0.1)	(3.3–3.3)	
5 mL			
Large end of the double-dosing spoon	5.0 (±0.7)	(4.8–5.2)	<0.001
Oral syringe	6.5 (±0.2)	(6.4–6.5)	

^1^ Student *t*-test for paired samples.

**Table 4:** Average weight (*n* = 40), standard deviation (SD) and 95% confidence interval (95% CI) for 2.5 and 5 mL of antibiotic A, measured with double-dosing spoon and B, measured with transparent dosing spoon.

Measured Volume	Average Weight (±SD) (g) *n* = 3	CI 95%	*p* ^1^
2.5 mL			
Small end of the double-dosing spoon	2.3 (±0.4)	(2.1–2.4)	<0.001
Transparent dosing spoon	3.4 (±0.4)	(3.3–3.6)	
5 mL			
Large end of the double-dosing spoon	5.0 (±0.7)	(4.8–5.2)	<0.001
Transparent dosing spoon	7.6 (±1.1)	(7.2–7.9)	

^1^ Student *t*-test for paired samples.

## Data Availability

Not applicable.
